# Quantitative association of blood culture volume with contaminant and pathogen recovery

**DOI:** 10.1016/j.imj.2026.100241

**Published:** 2026-02-23

**Authors:** Amos Cahan, Nadav Sorek, Tal Brosh-Nissimov

**Affiliations:** a Infectious Diseases and Infection Control Unit, Samson Assuta Ashdod University Hospital, Ashdod 7747629, Israel; b Clinical Microbiology Laboratory, Samson Assuta Ashdod University Hospital, Ashdod 7747629, Israel; c Faculty of Health Sciences, Ben Gurion University in the Negev, Beer Sheba 8499000, Israel

**Keywords:** Blood culture, Contamination, Commensal, Sample blood volume, Bacteremia

## Abstract

**Background:**

Blood culture contamination is common and associated with adverse outcomes. The relation between blood culture volume and contamination remains under-evaluated. We assess the relation between blood culture volume and commensal-positive culture rate and characterize the relative abundance of contaminant species in low- and high-volume samples.

**Methods:**

Analysis of blood cultures processed between August 2017 and August 2023 in an academic community hospital. Blood culture bottles containing 0–10 mL of fluid were included. Clinical data was not available. Contaminants were defined according to the National Healthcare Safety Network list of common commensals. The association between blood volume categories and the proportion of positive cultures was assessed using a linear logistic regression model. Goodness of fit was evaluated by restricted cubic splines and likelihood ratios.

**Results:**

Of a total of 146,307 blood culture bottles, 8,918 were excluded. A positive relation between blood volume and culture-positivity rate was found for both pathogens and contaminants, with an increase in odds ratio for culture positivity of 1.033 (95% CI: 1.026–1.041, *p* < 0.001) and 1.030 (95% CI: 1.018–1.041, *p* < 0.001) for each additional 1 mL for pathogens and contaminants, respectively. This translates to a 10 per 1000 excess cultures growing contaminants comparing 10 to 2 mL bottles. *Cutibacterium* was over-represented in high-volume samples. Otherwise, differences in relative abundance between the two volume categories were nonsignificant.

**Conclusions:**

The positive association between contaminant growth and blood volume may result from better growth conditions in high volume samples, or suggest that in some cases, contaminant growth may reflect frank bacteremia.

## Highlights

• We found higher sample blood volume to be associated with culture positivity.
• This was true not only for pathogens, as published, but also for contaminants.
• *Cutibacterium* species were more prevalent in high-volume samples.
• Findings question attributing contaminants in blood solely to the patient's skin.

## Graphical abstract

**Figure:**
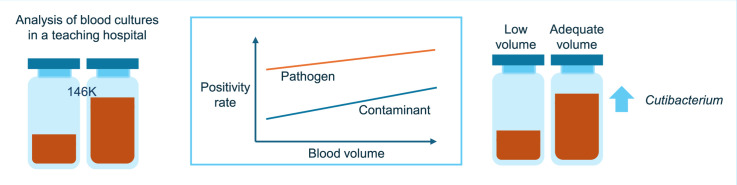

## 1 Introduction

The clinical significance of positive blood cultures (BC) varies greatly; a positive blood culture may indicate the presence of a life-threatening infection, yet frequently it is the result of contamination by exogenous microorganisms. Clinical guidelines recommend obtaining 2–3 blood culture sets, each comprising of 2 bottles with 8–10 mL of blood per bottle or 40–60 mL in total.^1^^,^^2^ Between 30% and 50% of positive blood cultures recover contaminants.^3^ Contaminated BC are associated with adverse outcomes, including unnecessary antibiotics, additional testing, unnecessary removal of vascular catheters, and longer admission.^3^ The cost of a contaminated blood culture has been estimated at $4500.^4^

The relation between sample blood volume and blood culture positivity is not adequately defined, with an association between the two reported by some studies but not others. To the best of our knowledge, there are no reports of a direct assessment of the relation between blood culture volume and contamination rate.

In this study, we aimed to assess the relation between blood culture volume and the rate of commensal growth and characterize the relative abundance of contaminant species in adequate volume (8–10 mL) and lower-volume BC samples.

## 2 Materials and methods

### 2.1 Blood culture processing and selection

All BC processed between August 2017 and August 2023 in the microbiology laboratory of the Samson Assuta Ashdod Hospital, an academic community hospital, were considered for inclusion. Blood cultures were processed by the BacT/Alert system (bioMérieux, Inc., Durham, NC, USA). The recommended blood volume to be filled in these bottles is 8–10 mL. Blood from bottles flagged as positive was Gram-stained and microorganisms identified using matrix assisted laser desorption ionization-time of flight mass spectrometry (MALDI-TOF MS; bioMérieux, Inc., Durham, NC , USA) and VITEK® 2 system (bioMérieux, Inc., Durham, NC , USA). Only one microorganism species per bottle (the first reported) was used in the analysis. Excluded from the analysis were cultures of pediatric blood culture bottles and bottles for which volume was not automatically recorded by the incubator. In some cases, this occurs due to a sticker attached to a bottle obscuring volume measurement. In others, volume may have been smaller than could be measured by the device. Blood culture bottles containing more than 10 mL of fluid were also excluded as they were not in line with manufacturer recommendations. Positive bottles in which the volume recorded by the incubator was 0 mL were included and assumed to contain a minute amount of blood. Contaminants were defined as species included in the National Healthcare Safety Network (NHSN) list of common commensals.^5^ This list includes species that may arguably be considered pathogens. We therefore performed a sensitivity analysis using a modified list excluding *Staphylococcus lugdonensis* and certain streptococci (*S. anginosus, S. constellatus, S. gallolyticus, S. gordonii, S. infantarius, S. intermedius, S. mutans* and *S. sanguinis*). An institutional board review assessment of this study was waived as it includes no patient data.

### 2.2 Statistical analysis

Analysis was done at the blood culture bottle level. Because our data did not include patient identifiers, we could not cluster multiple blood culture bottles taken from the same patient. We calculated the percentage of all positive BC and the percentage of all BC obtained that grow contaminants. Blood culture positivity rates were computed for 2 mL sample blood volume categories. A linear logistic regression model was fitted to the relation between blood volume and the rate of cultures growing contaminants. The exponentiated coefficient for blood volume was interpreted as the odds ratio (OR) for positivity for each 1 mL increase in blood volume. Absolute probabilities for positivity with 95% confidence intervals (CI) were computed for reference blood volumes (2, 4, 6, 8, 10 mL). To assess the fit of the linear models, we used restricted cubic splines. Goodness of fit was evaluated by likelihood ratio test.

We compared the abundance of bacteria between adequate volume as per blood culture bottle manufacturer specifications (≥ 8 and ≤ 10 mL) and low volume (< 8 mL) bottles. Relative abundance of bacteria was assessed at the genus level using Proportions-*Z*-test or Fisher's exact test, as appropriate. Results were corrected for multiple comparisons using the Holm-Bonferroni method. R version 4.4.3^6^ and R packages tidyverse^7^ and phyloseq^8^ were used for statistical analysis.

## 3 Results

During the study period, there were a total of 146,307 blood culture bottles analyzed by the microbiology laboratory. Of these, 8,918 were excluded as their volume was unmeasurable or exceeded 10 mL. Of the remaining 137,389 bottles, the mean blood volume was 4.4 mL (median 4.0 mL; inter-quartile range [IQR] 3.0–6.0 mL). Positive cultures were noted in 14,145 (10.3%), with 6,090 (4.4%; 43.0% of positive cultures) of them growing contaminants. The mean volume of positive bottles and of those growing contaminants was the same: 4.6 mL (median 5.0 mL; IQR 3.0–6.0 mL). Supplementary Table S1 lists the contaminant species isolated and their frequencies. Only the first species identified was included in the analysis in the case of 2,841 (21% of positive cultures) bottles growing more than one species.

**Fig. 1:**
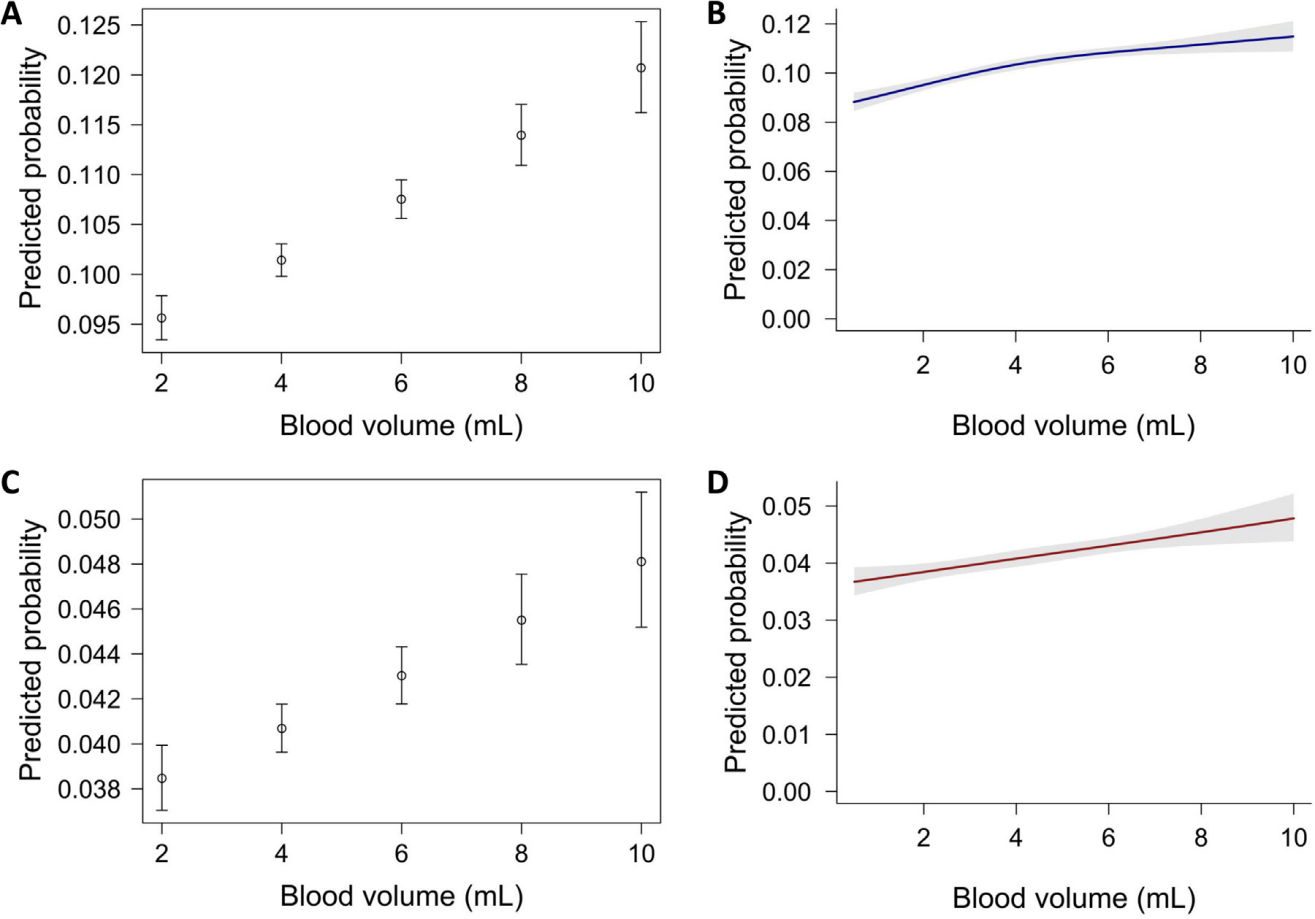
Association between blood volume and bacterial growth. (A) Linear logistic regression of blood culture positivity by volume; (B) Restricted cubic spline of blood culture positivity by volume; (C) Linear logistic regression of contaminant growth by blood volume; (D) Restricted cubic spline of contaminant growth by blood volume.

Using a linear logistic regression model, larger blood culture volumes were associated with higher odds for positivity, from 9.6% at 2 mL to 12.1% at 10 mL, with an OR for positivity of 1.033 (95% CI: 1.026–1.041, *p* < 0.001). Likewise, contaminant growth was positively associated with blood volume, with a predicted probability for positivity ranging from 3.8% at 2 mL to 4.8% at 10 mL, with an OR of 1.030 (95% CI: 1.018–1.041, *p* < 0.001) (Fig. 1). This corresponds to approximately 10 additional positive blood per 1000 cultures growing contaminants when volume is 10 mL compared to 2 mL.

Modeling using a restricted cubic spline (with an internal knot at the median and boundary knots at volume percentiles 10 and 90), the association between blood volume and positivity was mildly nonlinear with a flattening of the slope above 6 mL. The predicted probabilities for positivity were 9.5% (95% CI: 9.3–9.7%) and 11.5 (95%CI: 10.9–12.1%) at 2 mL and 10 mL, respectively (Fig. 1). A likelihood ratio test showed improvement over the linear model (∆Deviance = 6.69, *p* = 0.010). A spline model for contaminant growth yielded probabilities similar to the linear model (3.8% and 4.8% for 2 mL and 10 mL, respectively), with no improvement over the linear model (∆Deviance = 0.03, *p* = 0.850) (Fig. 1).

We next compared the relative abundance of contaminant bacterial species between adequate- (8–10 mL) and low- (< 8 mL) volume bottles. There were 642 adequate-volume and 5448 low-volume samples growing contaminants. Looking at relative abundance of contaminants in positive samples, staphylococci were under-represented in adequate-volume samples (78% vs. 82%), but the difference did not reach statistical significance. *Cutibacterium* (formerly *Propionibacterium*) was nearly 15 times more likely to be isolated from adequate-volume samples (relative abundance 5.6% vs. 0.4%, *p* < 0.001). Otherwise, differences in relative abundance between the two volume categories were nonsignificant (Table 1 and Supplementary Fig. S1), but the small numbers of some genera limited detecting differences. With a sensitivity analysis using the modified NHSN list, there were again no statistically significant differences in relative abundance except for *Cutibacterium* (data not shown).

**Table 1:** Relative abundance of contaminants in adequate- and low-volume bottles.

Genus	Total	Low volume (< 8 mL)		Adequate volume (8–10 mL)		*p*
		*N*	Proportion	*N*	Proportion	
Total	6090	5448		642		
*Actinomyces*	35	33	0.943	2	0.057	0.577
*Aerococcus*	1	1	1.000	0	0	1.000
*Alloiococcus*	4	4	1.000	0	0	1.000
*Arcanobacterium*	1	1	1.000	0	0	1.000
*Arthrobacter*	1	0	0	1	1.000	0.105
*Bacillus*	51	47	0.922	4	0.078	0.652
*Brevibacillus*	2	2	1.000	0	0	1.000
*Brevibacterium*	14	9	0.643	5	0.357	0.011*
*Cellulosimicrobium*	1	1	1.000	0	0	1.000
*Corynebacterium*	168	154	0.917	14	0.083	0.344
*Cutibacterium*	57	21	0.368	36	0.632	< 0.001
*Dermabacter*	20	20	1.000	0	0	0.260
*Exiguobacterium*	1	1	1.000	0	0	1.000
*Kocuria*	12	11	0.917	1	0.083	1.000
*Kytococcus*	1	1	1.000	0	0	1.000
*Micrococcus*	208	189	0.909	19	0.091	0.501
*Niallia*	2	2	1.000	0	0	1.000
*Paenibacillus*	5	4	0.800	1	0.200	0.427
*Rhodococcus*	2	2	1.000	0	0	1.000
*Rothia*	23	21	0.913	2	0.087	1.000
*Soliobacillus*	1	1	1.000	0	0	1.000
*Staphylococcus*	4958	4459	0.899	499	0.101	0.011*
*Streptococcus*	522	464	0.889	58	0.111	0.658

* Nonsignificant after Holm-Bonferroni correction.

## 4 Discussion

We analyzed over 130,000 BC bottles which were processed in the microbiology laboratory of a single teaching hospital over a period of 6 years. Overall, 10.3% of cultures were positive, with contaminants constituting 43.0%, which is in the range cited in the literature.

We found that the volume of blood in BC bottles was associated with the rate of positivity in a slightly nonlinear fashion, with a flattening of the slope above a volume of about 6 mL. These results are in line with those of other studies which found that the rate of positive BC is positively associated with blood culture volume. A study applying a generalized linear mixed model reported an OR of 1.13 for positivity for each additional milliliter of blood sampled.^9^ Another study reported the results of a multivariate model on BC obtained from a population of septic patients. The adjusted OR was 1.02, i.e. 2% increase in odds of positivity per each additional milliliter of blood.^10^ However, other studies could not show a relation between blood volume and positivity rate. In a study by Bouza et al.,^11^ blood volume was not an independent predictor of positivity, except among patients with high Acute Physiology and Chronic Health Evaluation II scores. A recent study by Romann et al.^12^ found no association between culture blood volume and the rate of positivity. These varying results may stem, in part, from the confounding factors, such as blood collection technique and different definitions of contaminants.

In the case of contaminants, we found a positive, linear association between blood volume and growth in culture, with an OR for positivity of (95% CI: 1.018–1.041, *p* < 0.001) per each additional mL. This translates to an estimated 10 per 1000 additional positive cultures growing contaminants for a culture blood volume of 10 mL, compared to 2 mL.

Since almost all contamination of BC is said to occur during blood collection,^2^^,^^13^ contaminants residing in the skin would be inoculated with the first milliliter of blood drawn, and thus one would expect a stable rate of growth in samples with different volumes. However, there are reports associating a higher rate of contamination with lower blood culture volume. In one study, the mean blood volume in positive cultures growing clinically relevant microorganisms was significantly larger than bottles with contaminants: 9.17 mL vs. 7.62 mL, respectively.^9^ Authors suggested that lower numbers of clinically relevant microorganisms found in samples with small blood volumes may remain undetected due to overgrowth of contaminants in the BC bottle. A retrospective analysis of 843 positive BC in pediatric patients found that of contaminants, 35% grew in adequate volume samples, compared to 60% in the true bacteremia group.^14^ Authors hypothesized that low-blood-volume cultures could have been drawn from patients with poor peripheral venous access, leading to compromise in disinfection technique during venipuncture.^14^ An alternative hypothesis may be that lower volume samples may reflect poor adherence with guidelines of the person obtaining the samples, which may also be a proxy for poor adherence with skin disinfection technique.

The finding that the rate of BC growing contaminants is positively associated with blood volume is thus surprising. One possible explanation to this finding relates to growth conditions inside BC bottles. These bottles contain nutrients, minerals and buffers that are designed to be diluted by blood to reach concentrations optimized for supporting bacterial growth. The addition to bottles of less than the recommended volume of blood might result in suboptimal growth conditions. Thus, a comparable inoculum of skin bacteria incubated in adequately filled bottles may hypothetically grow better than in under-filled bottles. The hypothesis that blood-to-broth ratio has bearing on bacterial growth was not supported in a study in contemporary blood culture systems and media. In the study, BC bottles were inoculated with standardized inoculums of several bacterial species after the addition of packed red blood cells (PRBC) in various volumes. No major differences were found in detection rates and time to positivity between PRBC volume groups across 8 common bacterial species causing bacteremia and 3 blood culture systems.^15^

An entirely different explanation of our finding might be that in some cases, isolation of contaminant species from blood reflects frank bacteremia. *Cutibacterium* species are part of the normal skin microbiota but may also be found in the oral cavity and large intestine.^16^ There is some evidence to support the view that it may also be a pathogen causing bacteremia. In a retrospective analysis of 816 *Cutibacterium* isolates, only 94 (12%) were deemed clinically significant and among patients with *Cutibacterium* infection, bacteremia was infrequent (16%).^16^ Another analysis of 522 patients with *C. acnes* bacteremia considered only 18 (3.5%) cases to be clinically significant (defined as isolation of *C. acnes* from 2 separate sets on the same day in the presence of an otherwise unexplained systemic inflammatory response syndrome).^17^ However, a higher rate (16%) of clinically significant bacteremia was reported in a recently published retrospective analysis of over 300 cases of blood cultures growing *C. acnes,* albeit using a slightly more relaxed definition of clinical significance.^18^ We found *Cutibacterium* to be nearly 15-fold more common in adequate-volume samples, supporting its role as a true pathogen.

Coagulase-negative staphylococci (CoNS) are commensals of the skin and common contaminants of blood cultures.^5^^,^^19^ We found that CoNS constitute a smaller proportion of contaminants in high volume samples, however this difference was not statistically significant. CoNS are the most commonly isolated contaminants, albeit isolation of CoNS from blood may reflect true infection, especially in the patients with central vascular catheters and implanted material such as prosthetic heart valves and joints. Indeed, in a retrospective analysis of 703 CoNS isolates from blood, 87 (12%) were considered true pathogens.^20^ The flip side of this finding is that other genera are represented more amongst high volume samples. As seen with pathogens, this could potentially be accounted for by increased odds of inoculating circulating bacteria with a larger sample volume. Differences between volume categories in the relative abundance of other genera were not statistically significant. The low absolute numbers were small, limiting discussion of species-level abundance.

This is an observational study, not allowing for causality to be established as to the relation between BC volume and positivity. The study assessed only BC with measurable volume; however we do not think this is likely to introduce bias to the interpretation of our results. In cultures with mixed growth, only the first reported species was included. This may lead to under-representation in the dataset of species growing in these cultures, which are more likely to be contaminants. Our study was limited by the fact that the dataset included only microbiologic data and did not include any patient data, preventing assessment of clinical correlates (and confounders) of blood culture positivity, or clustering cultures by patients. Lacking clinical data, computation of a contamination rate was not possible in this study. Moreover, we could not detect cases in which repeated BC were positive, which may have reduced variability and might have resulted in overestimated statistical significance. Severely ill patients are likely to have more blood cultures taken and are more likely to be immunocompromised. Thus, opportunistic, low-virulence pathogens may be over-represented in our dataset. The clinical importance of positive blood cultures growing low-virulence species depends on the presence of foreign material in the patient's body (e.g. prosthetic valve or joint). As mentioned, this information was not part of this study, limiting the interpretation the clinical importance of positive cultures. The source of sample, i.e. a peripheral vein or central vascular catheter, was also not available. Cultures taken from more severely ill patients are more likely to be drawn from a central line. Since there is usually no technical difficulty in withdrawing an adequate amount of blood from a central venous catheter (CVC), BC taken from CVC could be over-represented in the high-volume group. Colonization of central lines by low-virulence bacteria, reflected in some positive blood cultures, as well as central line related blood stream infections, may further lead to bias which cannot be accounted for in this study. Nevertheless, our institutional phlebotomy protocols limit BC to be drawn from central lines only during insertion. As noted, in the case of many of the contaminant species, the number of isolates was very small, limiting the assessment of species-level differences in relative abundance across volume categories. We used the NHSN list of common commensals, constituting a relaxed definition of species considered as contaminants (for instance, compared to a list issued by the American Society of Microbiology^21^). Nevertheless, well-known contaminants, such as *Pseudomonas stutzeri*, are not listed as commensals on that list. On the other hand, the list includes organisms which arguably should be considered pathogens, such as *S. lugdonensis*, the milleri-anginosus group of viridans streptococci, and others. As noted, a sensitivity analysis treating those as pathogens did not substantially impact our results. However, there is no single agreed upon list of contaminants, and analysis of the data using another list could have potentially yielded different results.

## 5 Conclusions

In this study, we add to the evidence associating higher blood volume in BC with the rate of BC positivity. The finding that this is true not only for pathogens, as previously described, but also for contaminants, questions the paradigm attributing contaminant growth in blood solely to bacteria found on the patient skin, and suggests that in some instances, microorganisms traditionally classified as contaminants might represent true bacteremia (or, less likely, promoted growth in higher-volume samples due to favorable culture conditions).

Our findings may have implications for clinicians and for laboratory work. It may be prudent for clinicians to cautiously interpret positive BC growing *Cutibacterium* based on the clinical setting. Clinical microbiology laboratories, when reporting positive as well as negative results, may consider annotating the sample volume, to help clinicians interpret results in conjunction with the patient's clinical context. Hospital infection prevention teams, when calculating contamination rates, may consider stratified analysis by sample volume to avoid bias due to differences in blood culture volume.

Further research based on larger datasets incorporating clinical correlates (e.g. patient immune status; catheter type; symptoms and signs of infection; laboratory infection markers) is required to validate the clinical significance of our observation that *Cutibacterium* growth is associated with BC volume. Future research could also characterize the relation between sample volume and growth of other species in blood cultures. It could also be assessed whether reducing the blood volume in BC may reduce the rate of cultures growing commensals. Yet, in current clinical practice, isolation of common contaminants from a single blood culture set should still be primarily considered contamination, unless supported by strong clinical or microbiological evidence (e.g., multiple positive sets, presence of an infectious focus, etc.).

## CRediT authorship contribution statement

**Amos Cahan:** Writing – original draft, Methodology, Formal analysis, Data curation, Conceptualization. **Nadav Sorek:** Writing – review & editing, Formal analysis, Data curation, Conceptualization. **Tal Brosh-Nissimov:** Writing – review & editing, Validation, Supervision.

## Informed consent

Informed consent was waived by the Assuta Ashdod Institutional Review Board.

## Organ donation

No blood donation samples were included in this study.

## Ethical statement

This study was conducted in accordance with the Declaration of Helsinki. The study protocol was reviewed and determined to be exempt by the Institutional Review Board (IRB) of Assuta Ashdod Hospital. The requirement for written informed consent was waived by the IRB due to the retrospective nature of the study and the use of de-identified laboratory data, which posed no risk to participants.

## Data availability statement

The dataset for this study is not publicly available due to institutional restrictions. However, de-identified data may be made available from the corresponding author upon reasonable request, subject to formal Institutional approval and the execution of a formal data use agreement.

## Animal treatment

Not applicable.

## Generative AI

During the statistical analysis of the data used in this manuscript, generative AI (ChatGPT [Open AI] and Perplexity [Perplexity AI]) were used for R script writing and interpretation of the results of statistical tests. The authors reviewed and edited the content as needed and take full responsibility for the content of the published article.

## Funding

None.

## Declaration of competing interests

The authors declare that they have no known competing financial interests or personal relationships that could have appeared to influence the work reported in this paper.

## Appendix Supplementary materials

Image, application 1
