# Supplementary material for: Quantitative association of blood culture volume with contaminant and pathogen recovery
Source: Infect Med (Beijing). 2026 Feb 23;5(1):100241. doi: 10.1016/j.imj.2026.100241 (PMC12966753; doi:10.1016/j.imj.2026.100241)
Supplement: Supplementary file 1 [file mmc1.docx]

**Supplementary**

**Supplementary Table S1: Frequencies of contaminant bacterial species.**

| Organism | Frequency |
| --- | --- |
| *Staphylococcus epidermidis* | 2149 |
| *Staphylococcus hominis* | 1484 |
| *Staphylococcus capitis* | 656 |
| *Staphylococcus haemolyticus* | 253 |
| *Micrococcus luteus* | 204 |
| *Streptococcus mitis/Streptococcus oralis* | 115 |
| *Staphylococcus hominis ssp hominis* | 95 |
| *Streptococcus gallolyticus ssp pasteurianus* | 82 |
| *Staphylococcus pettenkoferi* | 60 |
| *Staphylococcus caprae* | 58 |
| *Staphylococcus lugdunensis* | 57 |
| *Staphylococcus warneri* | 54 |
| *Streptococcus anginosus* | 52 |
| *Cutibacterium acnes* | 49 |
| *Streptococcus sanguinis* | 42 |
| *Streptococcus constellatus* | 41 |
| *Streptococcus salivarius ssp salivarius* | 41 |
| *Streptococcus gordonii* | 36 |
| *Corynebacterium striatum* | 32 |
| *Bacillus cereus group* | 28 |
| *Corynebacterium otitidis* | 28 |
| *Staphylococcus simulans* | 27 |
| *Streptococcus parasanguinis* | 23 |
| *Corynebacterium jeikeium* | 22 |
| *Streptococcus gallolyticus ssp gallolyticus* | 22 |
| *Staphylococcus auricularis* | 20 |
| *Staphylococcus ureilyticus* | 20 |
| *Corynebacterium aurimucosum* | 16 |
| *Dermabacter hominis* | 16 |
| *Streptococcus infantarius ssp coli (Str.lutetiensis)* | 16 |
| *Rothia kristinae* | 15 |
| *Streptococcus mitis* | 14 |
| *Aerococcus urinae* | 12 |
| *Staphylococcus saprophyticus* | 11 |
| *Actinomyces oris/viscosus* | 10 |
| *Bacillus licheniformis* | 10 |
| *Aerococcus viridans* | 9 |
| *Brevibacterium casei* | 9 |
| *Corynebacterium amycolatum* | 9 |
| *Corynebacterium pseudodiphtheriticum* | 9 |
| *Streptococcus mutans* | 9 |
| *Corynebacterium afermentans* | 8 |
| *Corynebacterium coyleae* | 8 |
| *Corynebacterium glucuronolyticum* | 8 |
| *Cutibacterium avidum* | 8 |
| *Kocuria rhizophila* | 8 |
| *Staphylococcus cohnii ssp cohnii* | 8 |
| *Bacillus subtilis/amyloliquefaciens/vallismortis* | 7 |
| *Corynebacterium durum* | 7 |
| *Streptococcus intermedius* | 7 |
| *Bacillus altitudinis/pumilus* | 6 |
| *Corynebacterium minutissimum* | 6 |
| *Staphylococcus pasteuri* | 6 |
| *Streptococcus salivarius ssp thermophilus* | 6 |
| *Actinomyces naeslundii* | 5 |
| *Arcanobacterium haemolyticum* | 5 |
| *Rothia dentocariosa* | 5 |
| *Streptococcus infantarius ssp infantarius* | 5 |
| *Paenibacillus provencensis* | 4 |
| *Brevibacterium luteolum* | 3 |
| *Corynebacterium mucifaciens/ureicelerivorans* | 3 |
| *Dermacoccus nishinomiyaensis/Kytococcus sedentarius* | 3 |
| *Micrococcus terreus* | 3 |
| *Rothia mucilaginosa* | 3 |
| *Staphylococcus saccharolyticus* | 3 |
| *Streptococcus constellatus ssp constellatus* | 3 |
| *Streptococcus vestibularis* | 3 |
| *Brevibacillus spp* | 2 |
| *Brevibacterium sanguinis* | 2 |
| *Corynebacterium imitans* | 2 |
| *Corynebacterium simulans* | 2 |
| *Corynebacterium urealyticum* | 2 |
| *Corynebacterium xerosis* | 2 |
| *Kocuria rosea* | 2 |
| *Paenibacillus lautus* | 2 |
| *Rhodococcus hoagii* | 2 |
| *Staphylococcus xylosus* | 2 |
| *Streptococcus alactolyticus* | 2 |
| *Aerococcus sanguinicola* | 1 |
| *Arthrobacter globiformis* | 1 |
| *Cellulosimicrobium cellulans* | 1 |
| *Corynebacterium freneyi* | 1 |
| *Corynebacterium kroppenstedtii* | 1 |
| *Corynebacterium mucifaciens* | 1 |
| *Corynebacterium tuberculostearicum* | 1 |
| *Dermacoccus nishinomiyaensis* | 1 |
| *Exiguobacterium aurantiacum* | 1 |
| *Kocuria palustris* | 1 |
| *Kocuria varians* | 1 |
| *Kytococcus schroeteri* | 1 |
| *Micrococcus lylae* | 1 |
| *Paenibacillus pueri* | 1 |
| *Solibacillus silvestris* | 1 |
| *Staphylococcus chromogenes* | 1 |
| *Staphylococcus equorum* | 1 |
| *Staphylococcus hominis ssp novobiosepticus* | 1 |
| *Staphylococcus succinus* | 1 |
| *Streptococcus cristatus* | 1 |
| *Streptococcus oralis* | 1 |
| *Streptococcus pluranimalium* | 1 |
| *Streptococcus thoraltensis* | 1 |
| *Trueperella bernardiae* | 1 |


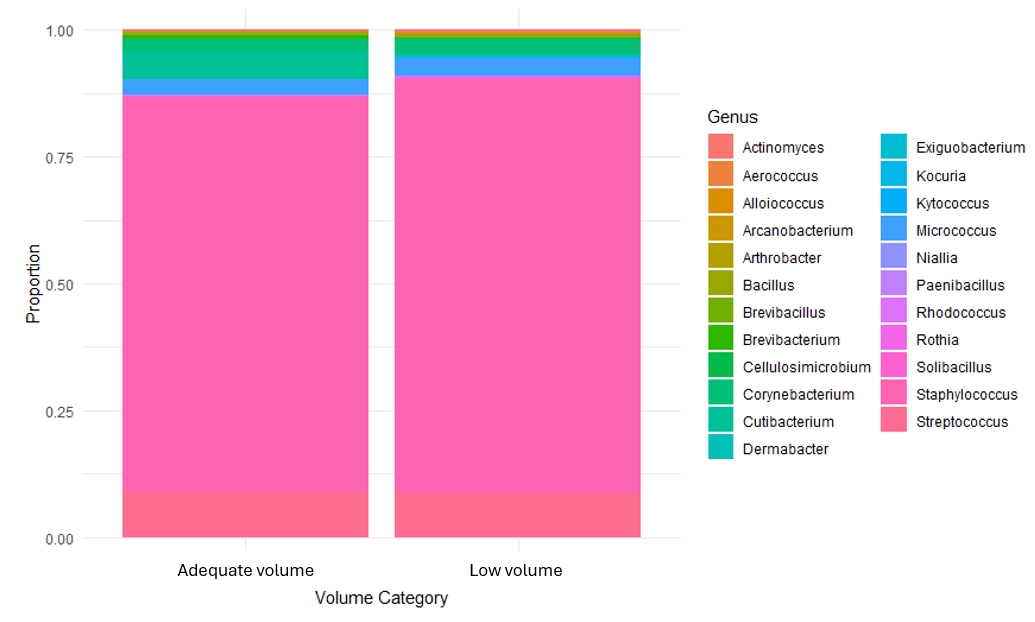


**Supplementary Fig. S1.** Relative abundance of contaminants by blood culture bottle volume. Adequate volume, 8–10 mL; Low volume, < 8 mL.
